# Re-stepping into the same river: competition problem rather than a reconsolidation failure in an established motor skill

**DOI:** 10.1038/s41598-017-09677-1

**Published:** 2017-08-24

**Authors:** Ella Gabitov, Arnaud Boutin, Basile Pinsard, Nitzan Censor, Stuart M. Fogel, Geneviève Albouy, Bradley R. King, Habib Benali, Julie Carrier, Leonardo G. Cohen, Avi Karni, Julien Doyon

**Affiliations:** 1 Department of Psychology, University of Montreal, Montreal, Quebec, H3C 3J7 Canada; 2 Functional Neuroimaging Unit, C.R.I.U.G.M., Montreal, Quebec, H3W 1W5 Canada; 3 Laboratory for Human Brain & Learning, Sagol Department of Neurobiology & the E.J. Safra Brain Research Center, University of Haifa, Haifa, 3498838 Israel; 4 Functional Neuroimaging Laboratory, INSERM U1146, Sorbonne University, Paris, 75634 France; 5 School of Psychological Sciences and Sagol School of Neuroscience, Tel Aviv University, Tel Aviv, 69978 Israel; 6 School of Psychology, University of Ottawa, Ottawa, Ontario, K1N 6N5 Canada; 7 Movement Control and Neuroplasticity Research Group, Department of Kinesiology, KU Leuven, Leuven, 3000 Belgium; 8 PERFORM Centre, Concordia University, Montreal, Quebec, H4B 1R6 Canada; 9 Human Cortical Physiology and Neurorehabilitation Section, National Institute of Neurological Disorders and Stroke, National Institutes of Health, Bethesda, Maryland 20892 USA

## Abstract

Animal models suggest that consolidated memories return to their labile state when reactivated and need to be restabilized through reconsolidation processes to persist. Consistent with this notion, post-reactivation pharmacological protein synthesis blockage results in mnemonic failure in hippocampus-dependent memories. It has been proposed that, in humans, post-reactivation experience with a competitive task can also interfere with memory restabilization. However, several studies failed to induce performance deficit implementing this approach. Moreover, even upon effective post-reactivation interference, hindered performance may rapidly recover, raising the possibility of a retrieval rather than a storage deficit. Here, to address these issues in procedural memory domain, we used new learning to interfere with restabilization of motor memory acquired through training on a sequence of finger movements. Only immediate post-reactivation interference was associated with the loss of post-training delayed gains in performance, a hallmark of motor sequence memory consolidation. We also demonstrate that such performance deficit more likely indicates a genuine memory impairment rather than a retrieval failure. However, the reconsolidation view on a reactivation-induced plasticity is not supported. Instead, our results are in line with the integration model according to which new knowledge acquired during the interfering experience, is integrated through its consolidation creating memory competition.

## Introduction

Compelling data from animal studies suggest that retrieval/reactivation of an existing memory trace may lead to its destabilisation^1, 2^, hence opening a limited time-window during which the memory can be degraded, maintained or enhanced through consolidation-like processes termed “reconsolidation”^3–5^. An important principle of the reconsolidation hypothesis is that post-retrieval pharmacological interference results in the loss of consolidated knowledge due to the disruption of the restabilization process of the memory trace. Most of the support for this idea comes from an amnesic phenomenon observed in animals using injection of protein synthesis inhibitors directly into specific brain areas in close temporal proximity to the reactivation of an established memory trace that hinders its restabilization. However, even such direct intervention may result only in transient impairments of performance with a high tendency for recovery^6, 7^, hence challenging the reconsolidation-based interpretation of the phenomena and raising the possibility of a failure in memory retrieval^8^ rather than memory trace decay^3^.

As protein synthesis inhibitors cannot be administered to humans, the introduction of a competing task, that has been shown to effectively block the consolidation process initiated by a novel learning experience^9–11^, has been adopted as a post-retrieval intervention in investigations of reconsolidation processes with human participants^9, 12–15^. For example, to test the susceptibility of previously consolidated procedural knowledge to post-retrieval interference, Walker *et al*.^9^ conducted a 3-day study of motor sequence learning using a computerized version of the sequential finger tapping task adapted from Karni *et al*. (1995, 1998). Training on a sequence (T-Seq) on Day1 was followed by the retest of the same sequence on Day2 to reactivate its memory trace. Immediately afterwards, participants were either trained, or not, on a second (novel) sequence. Finally on Day3, participants were retested again to assess changes as a result of the hypothesized post-reactivation reconsolidation process. As expected, post-training delayed gains in the performance of the T-Seq, a behavioral manifestation of motor sequence memory consolidation^9, 16–18^, were expressed during retest on Day2. Additional delayed gains in performance for the T-Seq were evident on Day3, but only if no post-reactivation interference in the form of training on the second movement sequence was afforded. The post-reactivation interference, on the other hand, resulted in a significant decrease in accuracy with no significant change in speed^9^. The latter result was interpreted as evidence for the notion that experience with a competing task may lead to the loss of consolidated knowledge disrupting a consolidated motor memory trace, presumably through interference with post-retrieval motor memory reconsolidation processes. However, in four replication attempts, some using software provided by Walker’s group, Hardwicke *et al*.^19^ failed to show any impairment in performance following post-retrieval interference in the form of new learning, thus failing to provide support for the reconsolidation theory.

Although conjectural, Hardwicke *et al*.’s (2016) replication failures could be due to boundary conditions in reconsolidation processes^19, 20^. For example, the length of the reactivation experience has been found to be a critical factor, such that a brief reactivation period renders the memory trace for a motor sequence labile and susceptible to interference, whereas a longer reactivation period may not^15^. Nevertheless, the detrimental effects of behavioral interference on the briefly reactivated motor task have been transient, with task performance rapidly recovered with continued practice^15^. The fast recovery of skilled performance challenges the concept of reconsolidation theory and suggests that the impairment following the post-reactivation interference procedure could be due to a transient failure in skill memory retrieval^8^ rather than its genuine degradation. Thus, whether, when and how a competing behavioral experience following the retrieval/reactivation of a given skill can interfere with reconsolidation processes and specifically degrade the pre-established “how to” knowledge remains an open question.

**Figure 1:**
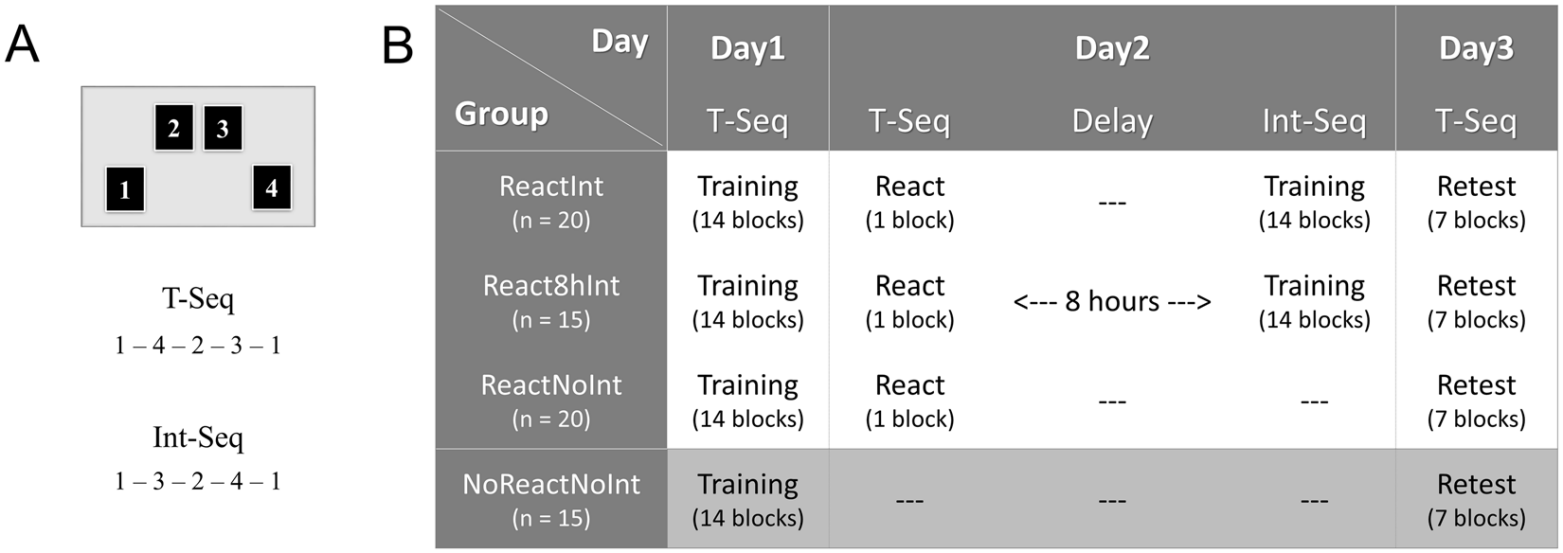
Study design. (**A**) Sequential finger tapping task. A sequence initially trained on the first experimental day (T-Seq, left panel) and a novel sequence used during the interfering training on the second day (Int-Seq, right panel). The two sequences were matched for number of movements per digit and mirror-reversed in relation to each other (in terms of order). (**B**) Experimental groups. On Day1, all groups underwent training on the T-Seq consisted of 14 performance blocks. On Day2, 55 participants underwent reactivation experience performing T-Seq (React, 1 block) and then were assigned either to an immediate interference group, an 8-hour interference group or a no interference group (ReactInt, React8hInt and ReactNoInt group, respectively). The interference was introduced behaviorally in a form of training on another (novel) sequence (Int-Seq). On Day3, performance for the T-Seq was tested in all groups using 7 performance blocks. In all sessions, performance blocks consisted of 60 key-presses, equivalent to 12 possible 5-element sequences, and were separated by 25-second periods of rest.

Using a 3-day motor sequence learning paradigm (Fig. 1) with a brief memory trace reactivation experience^15^, the current study provides strong evidence to the notion that behavioral interference can result in genuine impairment of a consolidated memory in humans. By manipulating the time between the motor memory retrieval and behavioral interference in a form of new learning, we first tested whether the susceptibility of the reactivated memory trace is limited to a time-window of a few hours duration, as previously shown for consolidation processes initiated by practice on a novel task^10, 11, 21^. To this end, three groups of participants were first trained on Day1, being instructed to tap a given sequence of finger movements (T-Seq) using a response pad. A single block performing the T-Seq was used on Day2 as a retrieval experience (i.e., reactivation), and the T-Seq was retested again on Day3. Behavioral interference, in the form of training on a second (novel) movement sequence (Int-Seq, composed of the same component movements but in a different order) was afforded to two (out of three) groups, either immediately or 8 hours after the brief experience with the T-Seq on Day2. To directly assess the impact of the brief post-training experience with the T-Seq afforded during reactivation on Day2 on its subsequent performance, a fourth group of participants was trained on Day1 and retested on Day3 only. We hypothesized that delayed consolidation gains would be expressed during a subsequent session in all groups (during the reactivation on Day2 in the first three groups or the retest on Day3 in the fourth group). Furthermore, we expected that these gains in performance would be negatively affected by immediate post-reactivation interference while performance levels following reactivation in two other groups (i.e., with 8-hour post-reactivation interference and without interference) would be preserved. Finally, it has been proposed that the difference in motor serial task representations is reflected in an actual tapping pattern of the sequence^22^. Therefore, we also expected that through analyses of patterns of inter-key press intervals, it would be possible to determine whether the loss of consolidation gains following effective post-reactivation interference reflect (or not) a genuine memory decay.

## Results

On Day1, all participants (4 groups, n = 70) underwent initial training on the sequential finger tapping task, which required subjects to tap repeatedly the assigned 5-element sequence of movements (T-Seq) using a response pad with the fingers of the left, non-dominant hand. To evaluate the effects of behavioral interference following the retrieval of the presumably consolidated T-Seq (reactivation) on Day2, 55 participants were randomly assigned either to an immediate interference, an 8-hour delayed interference or to a no interference condition (ReactInt group, n = 20; React8hInt group, n = 15; ReactNoInt group, n = 20, respectively). To control for the effects of the brief post-training experience with the T-Seq afforded on Day2 on its subsequent performance, 15 participants did not attend the second experimental day, and were thus asked to come back on Day3 only (NoReactNoInt group) (Fig. 1).

Motor skill performance was evaluated using a measure reflecting accuracy, i.e., a percentage of correct transitions, and a measure reflecting speed, i.e., the time (duration) per block spent at performing the task^23, 24^. To limit the period of memory trace reactivation as recommended by de Beukelaar *et al*.^15^, and to minimize the effects of the additional practice afforded in the retesting *per se*, performance level at each time-point of interest (i.e., initial learning, reactivation and retest) was evaluated using data from a single block corresponding to the execution of 12 consecutive 5-element sequences. Furthermore, to account for warm-up effects and an underestimation of the actual level of skill acquired^25^, performance measures were assessed using data from the final 30 key-presses (i.e., equivalent to 6 final sequences of the block^26^).

### Accuracy

**Figure 2:**
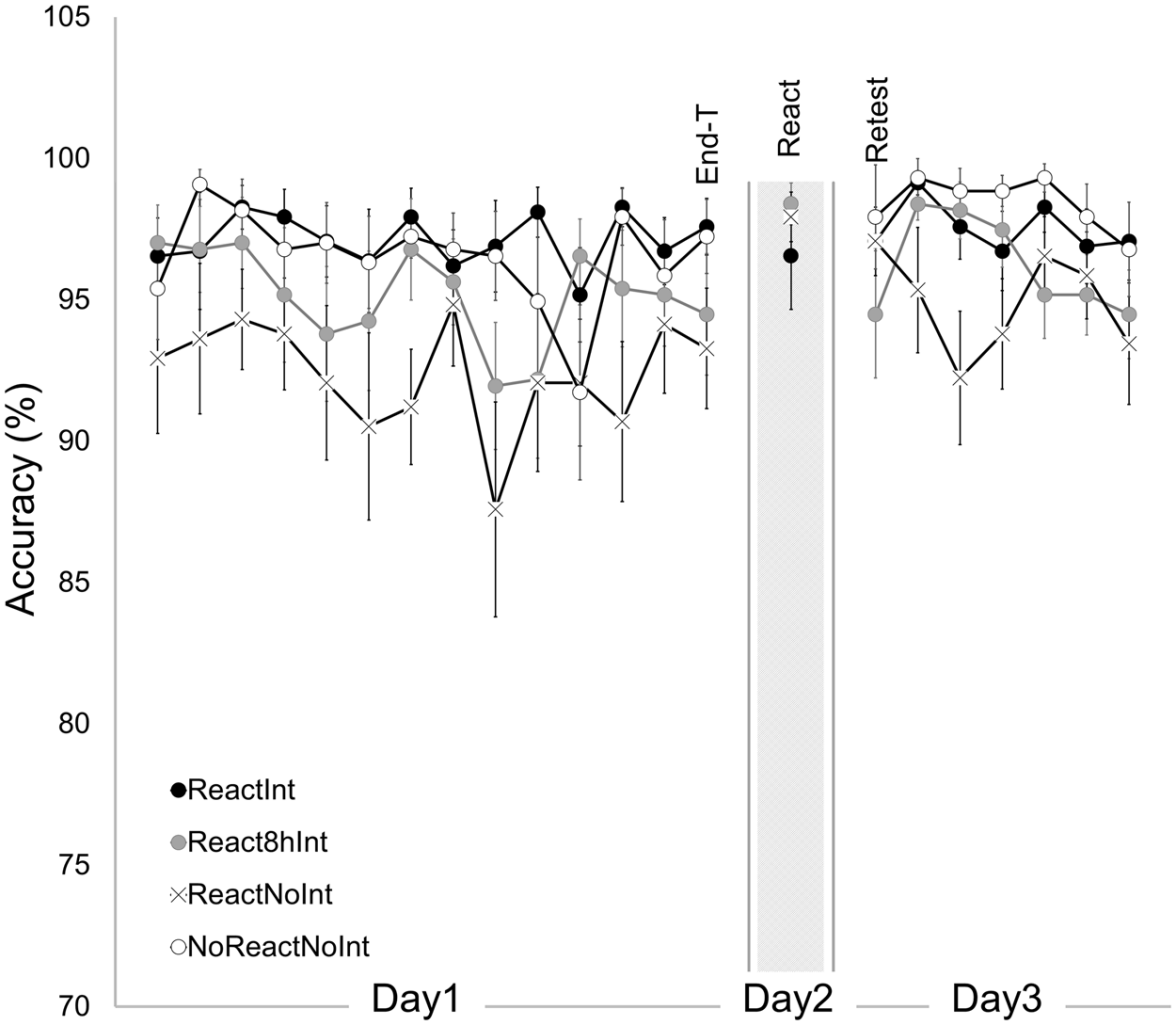
Accuracy. The percentage of correct transitions (i.e., transitions that correspond to 4 transitions between successive elements within a sequence, plus an additional transition between sequences; accuracy) for the last 30 key-presses for each performance block during the training, reactivation and retest (Day1, Day2 and Day3 respectively). Bars – standard error of the mean (s.e.m.).

Accuracy rates corresponding to the percentage of correct transitions (i.e., transitions that correspond to 4 transitions between successive elements within a sequence, plus an additional transition between sequences) are shown in Fig. 2. In line with previous reports^17, 18, 24, 27, 28^, performance on the task was characterized by high levels of accuracy in all groups (97.13 ± 1.07%; 95.16 ± 1.23%; 92.37 ± 1.07%; 96.50 ± 1.23%, mean ± s.e.m for ReactInt, React8hInt, ReactNoInt and NoReactNoInt respectively) with no significant improvement in accuracy rate during training on Day1. Repeated-measures ANOVA with *block* (14 training blocks) as a within-subject factor and *group* (ReactInt, React8hInt, ReactNoInt and NoReactNoInt) as a between-subject factor showed no significant effect of *block*, nor *block* by *group* interaction (*F*_(7.91,521.91)_ = 1.36, *p* = 0.21; *F*_(23.72, 521.91)_ = 0.69, *p* = 0.87, respectively), indicating that within-training gains in accuracy were absent in all experimental groups. There was, however, a significant *group* effect (*F*_(3,66)_ = 3.82, *p* = 0.01) that was driven by a significantly less accurate performance in the ReactNoInt group compared to the ReactInt and NoReactNoInt groups (p ≤ 0.01). Following consolidation interval, however, ReactNoInt group successfully closed this gap, hence being the only one (out of three groups that underwent reactivation on Day2) that showed significant delayed gains in accuracy as indicated by a t-test (two-tailed) comparing accuracy rates during the last training block on Day1 and the reactivation block on Day2 (*t*_(19)_ = 0.55, *p* = 0.59; *t*_(19)_ = −1.60, *p* = 0.13; *t*_(19)_ = −2.10, *p* < 0.05, ReactInt, React8hInt and ReactNoInt group respectively).

A repeated-measures ANOVA with *time-point* (End-T, React and Retest) as a within-subject factor and *group* (ReactInt, React8hInt and ReactNoInt) as a between-subject factor was used to test the effect of the post-reactivation interference experience. No significant results were derived from this analysis (*F*_(2,104)_ = 2.32, *p* = 0.10; *F*_(4,104)_ = 1.71, *p* = 0.15; *F*_(2,52)_ = 0.40, *p* = 0.67, *time-point* effect, *time-point* by *group* interaction and *group* effect respectively) indicating that overall, there were no significant changes in accuracy rate between the end of training on Day1, the reactivation block on Day2 and the retest on Day3 across groups. Moreover, the absence of significant *time-point* by *group* interaction also indicates that accuracy rates were not affected by different post-reactivation interference manipulations.

### Performance rate

#### Initial training

**Figure 3:**
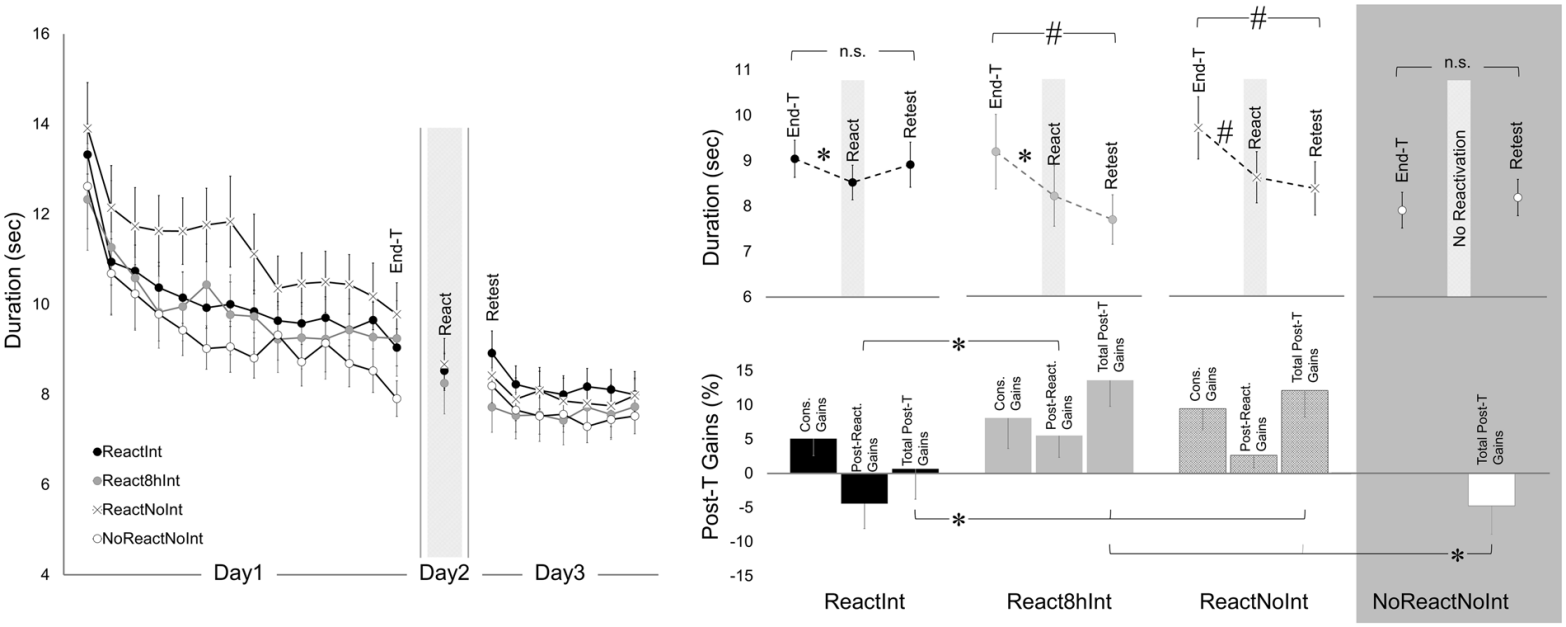
Performance duration and post-training gains. Time (i.e., duration) to complete the last 30 key-presses for each performance block during the training, reactivation and retest (Day1, Day2 and Day3 respectively) (left plot). Time to complete the last 30 key-presses during the last training-block, during the reactivation block and during the first retest-block (End-T, React and Retest respectively) (upper right plot). Gains in performance, normalized to the last training-block, that developed during the consolidation interval (i.e., between the end of training on Day1 and reactivation on Day2; Cons. Gains), after reactivation (i.e., between the reactivation on Day2 and retest on Day3; Post-React. Gains) and during the entire post-training period (i.e., between the end of training on Day1 and retest on Day3; Total Post-T Gains) (lower right plot). Note, positive values indicate performance improvements. Bars – standard error of the mean (s.e.m.). *Significant results at .05 level, ^#^Significant results at .01 level, n.s. – not significant.

Training resulted in a significant improvement of performance, with performance duration significantly shortened from 13.13 ± 0.46 sec to 9.05 ± 0.31 sec (mean ± s.e.m., initial and final training blocks, respectively) (*F*_(1,66)_ = 149.28; *p* < 0.001) with no significant group differences (*F*_(1,66)_ = .78; *p* = 0.51) (Fig. 3, left plot). Repeated-measures ANOVA with *block* (14 training blocks) as a within-subject factor and *group* (ReactInt, React8hInt, ReactNoInt and NoReactNoInt) as a between-subject factor showed significant effect of *block* (*F*_(5.32,351.41)_ = 35.87, *p* < 0.001), with no significant effect of *group* or *block* by *group* interaction (*F*_(3,66)_ = 1.44, *p* = 0.24; *F*_(15.97,351.41)_ = 0.78, *p* = 0.87, respectively), indicating robust improvement in performance over the course of initial training with similar performance levels across all groups.

#### Memory consolidation and time-dependent effect of interference on the reactivated motor memory trace

A single performance block used to reactivate the memory trace of the T-Seq on Day2 was completed in less than half a minute (17.81 ± 0.80 sec; 17.00 ± 1.37 sec; 18.13 ± 1.16 sec, mean block duration ± s.e.m. for the ReactInt, React8hInt and ReactNoInt group, respectively). Importantly, all three groups showed robust post-training delayed gains during this performance period, hence reflecting the expression of the procedural memory consolidation^9, 10, 17, 18, 29–32^. Overall, performance duration significantly reduced from 9.36 ± 0.38 sec during the last training block to 8.48 ± 0.32 sec during the reactivation block (mean ± s.e.m.; *F*_(1,52)_ = 22.60, *p* < 0.001) and did not differ between groups (*F*_(2,52)_ = 0.22, *p* = 0.80; *F*_(2,52)_ = 1.05, *p* = 0.36, effect of *group* and *time-point* by *group* interaction respectively) (Fig. 3, upper right plot).

A repeated-measures ANOVA with *time-point* (End-T, React and Retest) as a within-subject factor and *group* (ReactInt, React8hInt and ReactNoInt) as a between-subject factor was used to test the effect of the post-reactivation interference experience. There was a significant effect of *time-point* (*F*_(1.64,85.25)_ = 15.40, *p* < 0.001), indicating robust changes in performance between the end of training on Day1, the reactivation block on Day2 and the retest on Day3 across groups. Importantly, the *time-point* by *group* interaction was significant (*F*_(3.28,85.25)_ = 2.69, *p* < 0.05), suggesting that these changes in performance were modulated by the different post-reactivation interference manipulations. The consolidation gains expressed on Day2 by all groups (reported above) were degraded if interference training was afforded immediately following reactivation, so that performance in the ReactInt group during the first retest-block on Day3 did not differ from the performance achieved by the end of training on Day1 (*p* = 0.75) (Fig. 3). By contrast, the React8hInt group successfully carried over its consolidation gains to Day3 (*p* < 0.01), similar to the ReactNoInt group (*p* < 0.01). As a result, total post-training performance gains expressed on Day3 by the ReactInt group were significantly lower than in the React8hInt and ReactNoInt groups (*p* < 0.05), hence suggesting that new learning afforded one day after initial training on a motor sequence impaired performance of this sequence in a time-dependent manner (Fig. 3, lower right plot).

#### Effects of additional experience with the motor sequence task

Surprisingly, without any additional experience with the task during the post-training interval, no delayed gains in performance rate were evident by Day3 in NoReactNoInt group (paired t-test: *t*_(19)_ = −0.818, *p* = 0.427) (Fig. 3, right plots). Given that in the ReactNoInt group the post-training delayed gains in performance expressed by Day2 persisted, we assume that a brief reactivation experience alone could be an important factor in preserving consolidation gains keeping them readily available for longer term.

#### Fast performance improvement with continued practice in the absence of the post-training gains

**Figure 4:**
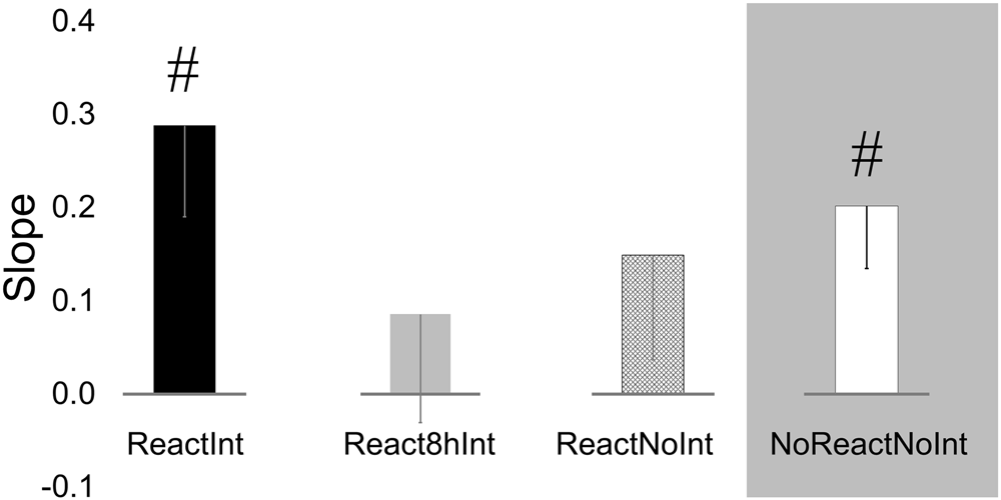
Fast performance improvement in the absence of post-training gains on Day3. Slopes, calculated as a gradient of linear regression line through 4 data points corresponded to the first 4 performance blocks during retest, are shown for each experimental group. Bars – standard error of the mean (s.e.m.). *Significant results at 0.05 level, ^#^significant results at 0.01 level. Note, positive slope coefficients indicate performance rate acceleration.

To gain insight into changes in performance with continued practice on Day3^15^, gradients of linear regression (slopes) through 4 data-points, corresponding to performance during the first 4 blocks of the retest on Day3 were calculated^33^ (Fig. 4). Abolishment of consolidation gains in the ReactInt group during the first retest-block on Day3 was associated with significant performance improvement during the subsequent blocks, as indicated by a t-test (two-tailed) performed on the slope coefficients (*t*_(19)_ = 2.93, *p* < 0.01). Similar effects were observed in the absence of experience with the T-Seq on Day2 (i.e., NoReactNoInt group, *t*_(14)_ = 3.02, *p* < 0.01). However, participants of the React8hInt and ReactNoInt group, who successfully carried over consolidation gains expressed on Day2 to Day3, did not show significant within-test improvement with continued practice. Similar pattern of results was evident taking into account data from all 7 blocks of the retest (*t*_(19)_ = 2.25, *p* < 0.05; *t*_(14)_ = −0.20, *p* = 0.85; *t*_*(19)*_ = 1.58, *p* = 0.13; *t*_*(14)*_ = 2.28, *p* < 0.05, ReactInt, React8hInt, ReactNoInt and NoReactNoInt group respectively). Thus, fast improvement in performance during the retest was associated with an initial failure to express post-training gains on the last experimental day.

### Dynamic changes in a pattern of inter-key press intervals

The question whether the deficit in post-training performance gains could result from a genuine loss of consolidated knowledge or from a retrieval failure was addressed by testing for experience-driven changes in the pattern of inter-key press intervals (IPIs)^28, 34^. IPIs pattern is a 5-element vector of time-intervals (i.e., durations) between key presses that correspond to 4 transitions between successive elements within a sequence, plus an additional transition between sequences (Supplementary Fig. S1). Changes in IPIs pattern driven by practice and post-training behavioral manipulations were assessed using Pearson correlation coefficients calculated between patterns at different time-points of interest. To allow for proper inferences, correlation coefficients were transformed to the standard normal z distribution using Fisher’s z-transformation. Note, that correlation coefficients indicate the degree of similarity between IPIs patterns (i.e., higher values corresponding to greater pattern similarity, and vice versa), but do not reflect changes in overall performance rate. Moreover, this approach takes into account the inter-subject differences in the way the IPIs pattern is formed and modified by previous experience with the task^28, 34, 35^ and allows to perform statistical inferences without making any assumption about the shape and experience-driven changes of the IPIs pattern on the group level.

**Figure 5:**
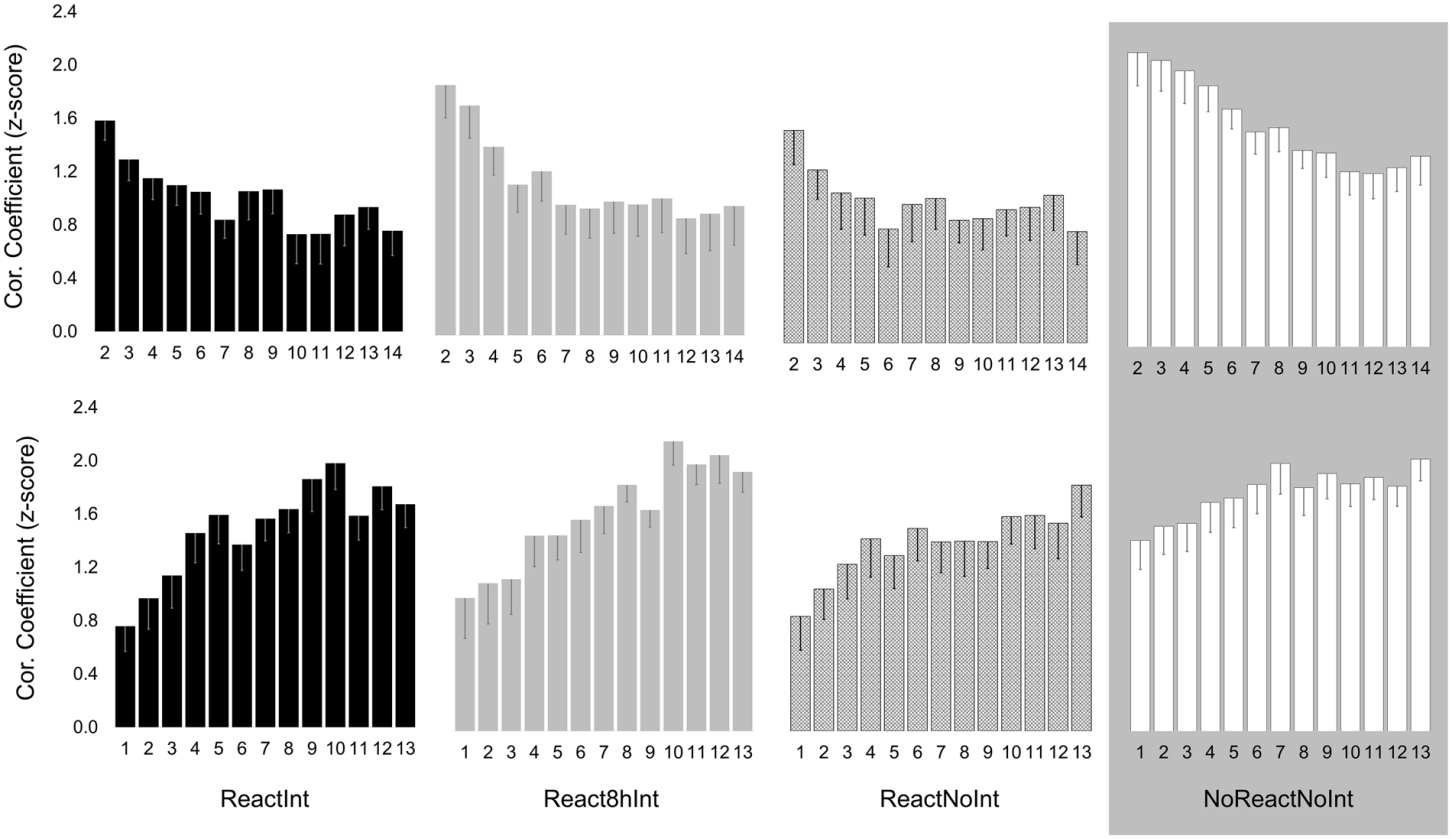
Dynamic changes in a pattern of inter-key press intervals during the initial training on Day1. Degree of similarity between patterns of inter-key press intervals (IPIs) was assessed based on normalized Pearson correlation coefficients calculated for each individual using the Fisher’s z-transformation. Mean normalized Pearson correlation coefficients between the primary IPIs pattern generated during the first training block and the subsequent practice (i.e., training blocks 2–14) for each group (upper plot). Mean normalized Pearson correlation coefficients between the IPIs pattern formed by the end of training (i.e., during the last training block) and the preceding practice (i.e., training blocks 1–13) for each group (lower plot). Bars – standard error of the mean (s.e.m.).

First, we examined whether and how the primary pattern of inter-key press intervals was modified by practice. Normalized Pearson correlation coefficients calculated in each group and for each training block versus the first and last training blocks on Day1 are shown in Fig. 5 (upper and lower plots respectively). During initial training, the IPIs pattern underwent significant changes across all groups so that the degree of similarity to the primary IPIs pattern (i.e., generated by each participant during the first training block) decreased significantly from 1.80 ± 0.11 to 0.99 ± 0.12 (mean ± s.e.m., correlation coefficients for the second and last training blocks, respectively) (*F*(1, 66) = 49.61; *p* < 0.001) while the degree of similarity to the IPIs pattern formed by the end of training (i.e., generated by each participant during the last training block) significantly increased from .99 ± 0.12 to 1.84 ± 0.10 (mean ± s.e.m., correlation coefficients for the first and the penultimate training block, respectively) (*F*(1, 66) = 70.70; *p* < 0.001). Thus, rapid and robust improvements in performance rate expressed by the end of the training were also associated with formation of a new, presumably more efficient pattern to generate the sequence. It is worth noting that the IPIs pattern formed by the end of training was different across participants and did not converge to the same shape (Supplementary Fig. S1).

**Figure 6:**
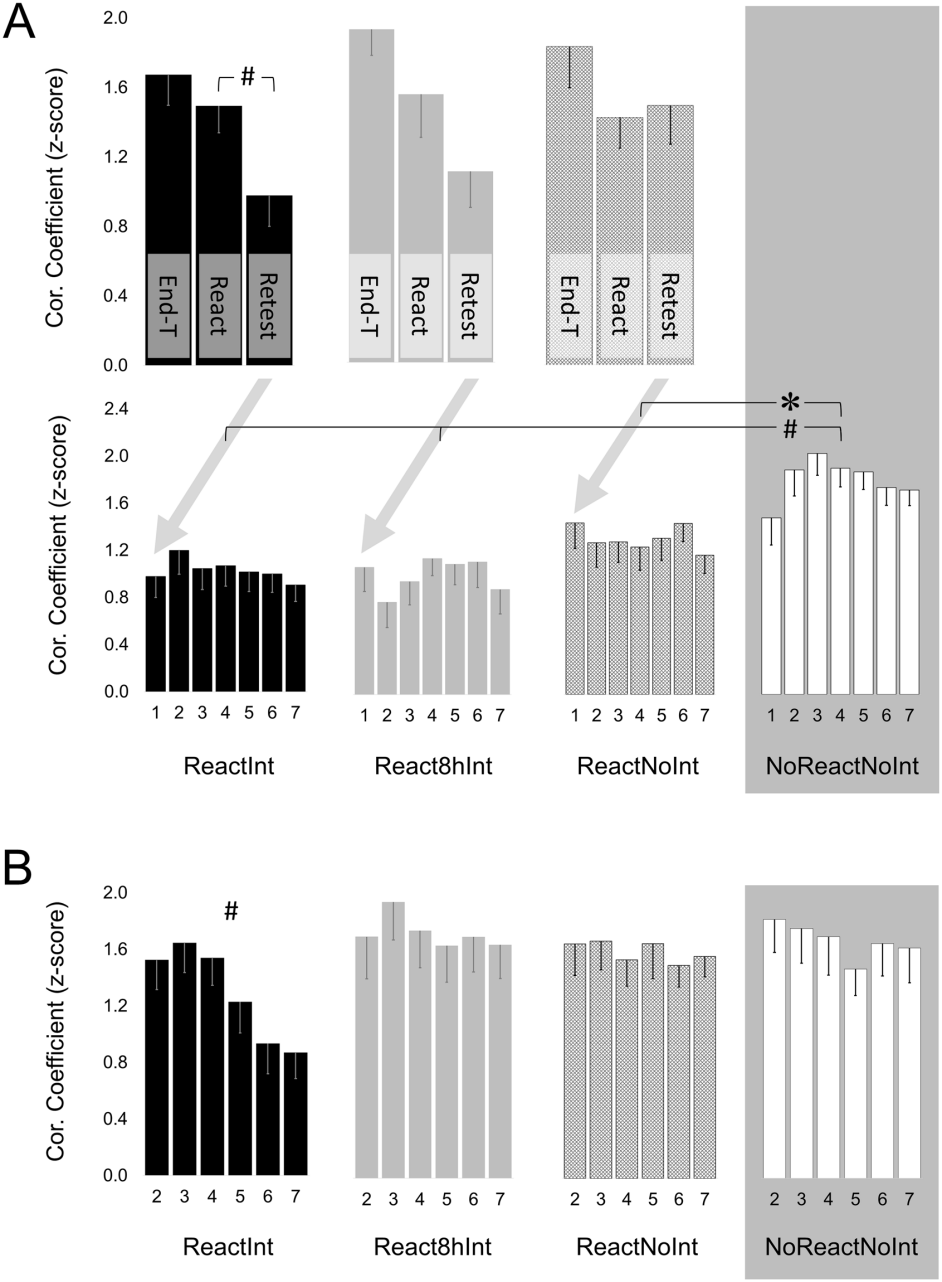
Degree of similarity between patterns of inter-key press intervals. Degree of similarity between patterns of inter-key press intervals (IPIs) was assessed based on normalized Pearson correlation coefficients calculated for each individual using the Fisher’s z-transformation. (**A**) Mean normalized Pearson correlation coefficients of IPIs pattern formed by the end of the initial training (i.e., during the last training block on Day1) with IPIs pattern for the reactivation block on Day2 (React) and for the first retest-block on Day3 (Retest) for ReactInt, React8hInt and ReactNoInt group (upper plot). Mean normalized Pearson correlation coefficients between IPIs patterns formed by the end of the initial training on Day1 and each retest-block on Day3 (1–7) for each group (lower plot). (**B**) Mean normalized Pearson correlation coefficients between IPIs patterns for the first retest-block and during the subsequent repeated practice (i.e., retest-blocks 2–7) for each group. Bars – standard error of the mean (s.e.m.). *Significant results at .05 level, ^#^Significant results at .01 level.

Following the consolidation phase, the IPIs pattern formed by the end of the initial training underwent additional modification, as indicated by its weaker correlation with the IPIs pattern observed during the reactivation block on Day2 (React) versus its correlation with the IPIs pattern observed during the penultimate training block on Day1 (End-T) (Fig. 6A, upper plot). The decrease in IPIs pattern similarity overnight was significant across all groups that underwent an actual reactivation of the memory trace for the T-Seq on Day 2 (*F*(1, 52) = 14.34, *p* < 0.001). This result is in line with the notion that the overnight consolidation of a motor skill involves changes in the motor sequence representation^28^. Yet, the correlation between IPIs patterns at the end of training and during the reactivation block remained strong (1.49 ± 0.16, 1.50 ± 0.24, 1.34 ± 0.17, mean ± s.e.m for ReactInt, React8hInt and ReactNoInt group respectively) and was significantly higher than the correlation between the IPIs patterns during the first and the last block of the initial training on Day1 across groups (*F*(1, 52) = 29.80, *p* < 0.001).

To assess changes in the pattern of inter-key press intervals following actual reactivation of the consolidated memory trace on the second day, normalized Pearson correlation coefficients resulted from correlation of the IPIs pattern during the reactivation block on Day2 (React) and during the first retest block on Day3 (Retest) with the IPIs pattern formed by the end of the initial training (i.e., during the last training block on Day1) were analysed (Fig. 6A, upper plot). The repeated-measures ANOVA with *time-point* (React and Retest) as a within-subject factor and *group* (ReactInt, React8hInt and ReactNoInt) as a between-subject factor showed significant effect of *time-point* with significant *time-point* by *group* interaction (*F*_(1,52)_ = 9.39, *p* < 0.01; *F*_(2,52)_ = 3.90, *p* < 0.05, effect of *time-point* and *group* by *time-point* interaction respectively). This result indicates that from Day2 to Day3, a degree of the IPIs pattern similarity with the one formed by the end of the initial training decreased across groups; this decrease, however, differed between groups as a function of reactivation-interference manipulation. Post hoc tests performed separately for each group showed significant decrease in the degree of pattern similarity only in the ReactInt group (*p* = 0.001; *p* = 0.11; *p* = 0.58, ReactInt, React8hInt and ReactNoInt group respectively).

Analyses of correlation coefficients calculated in each group for each retest-block, versus the last block of training on Day1, showed greater similarity between IPIs pattern during the final retest and IPIs pattern formed by the end of training in the NoReactNoInt group, compared to all other groups (significant *group* effect resulting from repeated-measures ANOVA: *F*_(3,66)_ = 5.08, *p* < 0.01, and post hoc t-tests: *p* < 0.05) (Fig. 6A, lower plot). This finding suggests that any experience with the task during the post-training interval modified the IPIs pattern implemented during the T-Seq performance at the final retest. Furthermore, similar analysis using data only from the first 4 retest-blocks resulted in significant *block* by *group* interaction (*F*_(7.67,168.80)_ = 2.85, *p* < 0.01). A post-hoc analysis performed separately for each group revealed a significant block effect for the NoReactNoInt group (*F*_(2.29,31.99)_ = 3.91, *p* < 0.05) so that correlation with IPIs pattern formed by the end of training increased across the first retest-blocks. As reported above, this rapid recovery of the IPIs pattern formed by the end of training during the retest was also associated with faster performance. Noteworthy, no recovery of previously implemented IPIs patterns, i.e., pattern either formed by the end of training or during the reactivation block, was evident in the other groups (ReactInt, React8hInt, ReactNoInt) (Fig. 6A, lower plot and Supplementary Fig. S2). We, thus, suggest that the increase in similarity between IPIs pattern during the retest on Day3 and IPIs pattern formed by the end of training on Day1, in the absence of any experience with the task in between (i.e., NoReactNoInt group), may reflect an initial failure in memory retrieval and its rapid rescue with continued practice. The greater reliance on previously formed IPIs pattern also allowed for rapid recovery of previously obtained motor skill levels. On the other hand, the rapid within-test improvement in performance rate observed in the ReactInt group was not characterized by recovery of previously formed IPIs patterns and, therefore, is unlikely to reflect the rapid rescue of previously established memory following an initial retrieval deficit.

Finally, we tested whether IPIs pattern during the first retest-block underwent significant changes with continued practice by comparing the degree of IPIs patterns similarity between the first and the remaining (i.e., 6 out of the 7) retest-blocks (Fig. 6B). Only following immediate post-reactivation interference, IPIs pattern generated at the very beginning of the retest session on Day3 was significantly modified by continued practice, as indicated by a significant effect of *block* across groups (*F*_(4.24,280.00)_ = 4.68, *p* = 0.001), a marginally significant *block* by *group* interaction (*F*_(12.73,280.00)_ = 1.70, *p* = 0.07) and a significant effect of *block* only in the ReactInt group in the subsequent post hoc analyses (*F*_(3.59,68.12)_ = 8.37, *p* < 0.001; *F*_(3.00,42.01)_ = 0.50, *p* = 0.68; *F*_(4.05,77.00)_ = 0.37, *p* = 0.83; *F*_(3.32,4646)_ = 1.56, *p* = 0.21, ReactInt, React8hInt, ReactNoInt and NoReactNoInt group respectively). Thus, the rapid within-test improvement in performance rate observed one day after effective post-reactivation interference did not result from a simple performance acceleration, but rather required or occurred due to significant changes in IPIs pattern. The fact that these changes were not associated with greater reliance on previously formed IPIs patterns more likely indicates that these patterns were not available/not accessible, as opposed to the NoReactNoInt group. Therefore, we propose that the impaired performance rate after the immediate post-reactivation interfering training reflects the genuine loss of consolidated knowledge. However, decreased similarity of IPIs pattern on Day3 to one formed by the end of the initial training on Day1, i.e., before consolidation processes of newly acquired skill took effect, indicates that rather than hindering memory trace restabilization, behavioral interference resulted in a genuine modification of the consolidated memory trace.

## Discussion

The goal of our study was to test whether, in humans, behavioral interference, in a form of new learning, can be used as a reliable method to disrupt a putative post-reactivation reconsolidation process of procedural (“how to”) motor memory leading to a genuine loss of the consolidated knowledge. To this end, we first examined the time-dependent effect of new learning, introduced after a brief reactivation experience on Day2, on the subsequent performance of the initially trained sequence on Day3. Only immediate post-reactivation experience with the Int-Seq, but not after 8-hour delay, was associated with degraded performance of the initially trained sequence so that post-training consolidation gains, expressed for the T-Seq by all groups on Day2, were lost. Furthermore, by assessing changes in the patterns of inter-key press intervals, we showed that under-expression of consolidation gains on Day3 following immediate post-reactivation interfering experience could not be explained by a transient retrieval failure, but more likely reflected genuine memory impairment. Moreover, the current study is the first one to provide insights into the nature of the consolidated memory loss induced by behavioral interference. Our findings are not in accord with the reconsolidation view on retrieval-induced plasticity and suggest instead that a competitive learning experience, juxtaposed to the retrieval of a pre-established movement routine, modifies the “how to” motor memory through integration of the new movement routine with the old one hence creating interference.

In the current study, only one performance block was used on Day2 to reactivate the previously established motor memory trace. While all three groups that took part in the reactivation phase expressed significant consolidation gains overnight during the reactivation block, this brief experience with the initially trained sequence *per se* did not contribute to further significant improvement in performance rate form Day2 to Day3. Yet, one reactivation block was sufficient to make the retrieved memory susceptible to behavioral interference. The detrimental effects of immediate post-reactivation interference were expressed in the loss of consolidation gains overnight. Our results, together with the negative outcome from the latest study by Hardwicke and colleagues (2016) who did not observe performance degradation from Day2 to Day3 following retest-interference manipulation using a very similar design but with longer reactivation phase, provide strong support to the notion that the length of reactivation is a crucial boundary condition determining whether consolidated motor memories in humans can be partly disrupted or not^15^. However, it does not imply that interference following longer reactivation experience used in previous studies has no effect on retrieval-induced plasticity. In the latter case, rather than inducing a loss of the previously consolidated knowledge, the post-reactivation interference could block additional delayed gains in performance that are typically observed overnight between Day2 and Day3 when no interference is afforded^9, 29, 30^. Thus, it may be the case that instead of hindering reconsolidation/restabilization processes for a previously consolidated motor memory trace, an interfering experience may affect the retrieved memory through consolidation processes initiated for the combined experience of old and new movement routines.

The detrimental effects of immediate post-reactivation interference reported by Walker *et al*.^9^ were expressed in a significant decrease in accuracy with no significant change in speed. However, the current results as well as the outcomes of other studies that used variations of similar paradigm, with either interference in a form of new learning or transcranial magnetic stimulation^15, 29, 30^, are not consistent with the former pattern of results and show, instead, that effective post-reactivation interference significantly affect subjects’ performance rate with no significant impact on accuracy. The null effect on accuracy can be explained by the fact that in the current study participants showed highly accurate performance from the very beginning of the experiment and, having no room for practice-induced improvement, it was also not possible to observe interference effects on accuracy. Noteworthy, studying other aspects of motor learning, it has been consistently shown that experience and memory processes for explicitly known motor sequences are associated with substantial changes in performance rate while the number of errors remains minimal^17, 18, 24, 27^.

In the current study, experience-driven changes in the pattern of inter-key press intervals were assessed using Pearson correlation coefficients between IPIs patterns at different time-points of interest calculated for each individual. This approach is advantageous to address inter-subject differences in the motor sequence pattern^28, 34, 35^ and allows to perform statistical inferences without making any assumption about the shape of the IPIs pattern and/or its modification at the group level. Moreover, this measure does not reflect changes in performance speed per se and may thus be more sensitive to changes in task representation within the neural system. However, in order to gain insight into the nature of underlying processes and their effect on memories, changes in IPIs pattern similarity should be considered together with changes in performance speed.

In line with the recent study^15^, the impaired performance observed for the ReactInt group on Day3 rapidly improved within the retest session. A similar performance deficit and rapid improvement with continuous practice were also observed in the NoReactNoInt group. However during retest on Day3, the ReactInt and NoReactNoInt groups were radically different in their IPIs pattern dynamics so that the fast recovery of the previously formed IPIs pattern was evident only in the latter, when no post-training experience with the task on Day2 was afforded. We conjecture that the performance deficit followed by a fast recovery of the previously formed IPIs pattern observed in the NoReactNoInt group presumably indicates an initial failure in memory retrieval. In this case, fast regain of performance rate with continuous practice presumably indicates that increased reliance on the neural network previously established to generate the trained sequence allowed to rapidly recover previously obtained motor skill levels. By contrast, we did not find any evidence for rescue of previously formed IPIs patterns, i.e., the one formed by the end of training on Day1 or during the reactivation block on Day2, in the ReactInt group. The latter result presumably indicates that the previously formed IPIs patterns were not available/not accessible following immediate post-reactivation interference. Thus, retrieval failure as a possible explanation for performance deficit on Day3 observed in the ReactInt group is not supported. Instead, we propose that the loss of consolidation gains induced by behavioral interference implies a genuine impairment of the motor skill memory. In this case, rapid improvement in performance with continuous practice, paralleled by progressive changes in IPIs pattern in the ReactInt group during the retest, may reflect memory encoding processes during which a new memory trace is routed. Indeed, characteristic changes in the IPIs pattern on Day3 following immediate post-reactivation interference were similar to those observed during the initial training so that the degree of similarity to the IPIs pattern generated by each participant during the first block progressively decreased with practice. It has been suggested that changes in IPIs pattern during sequence production reflect fundamental changes in its neural representation^22^, presumably implying the selection of neural ensembles to generate a sequence of movements in a more efficient way^36^. Such optimization processes would involve fluctuations between different solutions available within the redundant motor system and would either be driven by the goal to minimize the cost function associated with movement as proposed by the optimal control model^37^, or be guided by prior beliefs about the desired action upon active inference mechanism^38^.

If the loss of consolidation gains observed in the current study one day after immediate post-reactivation interference is the consequence of a restabilization failure^15^, one would have expected that the expression of the impaired memory would have been characterized by a greater reliance on task representation formed by the end of the initial training, i.e., before the newly encoded memory was stabilized through consolidation. However, our results indicate the opposite. Although overnight consolidation gains in performance were associated with significant changes in the IPIs pattern, the correlation between the IPIs pattern generated during the reactivation block on Day2 and the IPIs pattern formed by the end of the initial training on Day1 remains strong (Fig. 6A). From Day2 to Day3 this strong similarity to the IPIs pattern formed by the end of training significantly decreased but only if immediate post-reactivation interference was afforded (i.e., in the ReactInt group). Thus, strengthening and weakening of motor memory as indicated by consolidation gains overnight and their loss following effective post-reactivation interference, respectively, were associated with significant changes in the IPIs pattern so that in both cases its similarity to the IPIs pattern formed by the end of the initial training decreased. We suggest that, rather than a failure in restabilization/reconsolidation of the reactivated memory trace, the characteristic changes in IPIs pattern observed in the current study support a model where post-reactivation plasticity induced by behavioral intervention involves integration of new information through its consolidation^39^. According to this model, integration of new information depending on its content can either compete or not with the older memory resulting in degraded or improved performance^7, 40^.

It is worth noting that stable performance in participants who successfully carried over consolidation gains expressed during the reactivation block to the last experimental day was associated with stable IPIs pattern during the retest session. However, in these participants inferior similarity to the IPIs pattern formed by the end of training, compared to the NoReactNoInt group, suggests that any experience with the task modifies its representation. Here we show that performance rate as a measure of motor skill level alone may not be sensitive enough to these qualitative changes within the neural system. Obviously, the changes in task representation may be driven by various factors such as practice, off-line processes or interference, hence resulting in strengthening or weakening of previously established memories. However, further studies are required to better determine how particular characteristics of previous experience shape motor memories.

Despite great progress made in the elucidation of brain mechanisms involved in memory processes, the biological underpinnings of the time-dependent susceptibility to interference are still obscure. It has been suggested that interference by new learning afforded shortly after initial training may result from usurpation of resources that might otherwise be used to consolidate knowledge for the previously encoded motor sequence through mechanisms outlined in the synaptic tagging hypothesis^41^. The synaptic tagging hypothesis posits that synapses activated during learning are temporarily tagged so that plasticity-related proteins generated in the cell body are captured by those synapses^42^. Trafficking of plasticity-related proteins is essential for sustained long-term potentiation. Furthermore, synapses closer to those that are strongly stimulated and tagged during learning are more likely to be potentiated as well for a certain period of time. The induction of synaptic potentiation creates the potential for a lasting change in synaptic efficacy. One would thus assume that similar processes may be triggered by reactivation of the consolidated memory trace, hence increasing the probability that learning of a new sequence immediately after reactivation tags synapses located nearby to the reactivated ones. Consequently, the integration of different tasks representations in close proximity within the same population of neurons would create an interference problem^43^.

Taken together, our findings suggest that behavioral interference, in a form of new learning of competitive task juxtaposed to a brief reactivation experience, can lead to genuine degradation of the pre-established “how to” knowledge in humans. However, rather than inducing a restabilization process, reactivation of a consolidated motor sequence memory trace triggers consolidation processes allowing integration of new information with the old one and, hence, increasing the probability that learning of a new sequence immediately after reactivation will create an interference problem. The transient nature of interference-induced deficit in motor skill observed in the current study cannot be explained by rapid memory rescue following initial failure in retrieval. Instead, we assume that following effective interference, rapid improvement in performance with the continuous practice reflects memory encoding process during which a new memory trace is routed.

## Methods

### Ethics Statement

All participants gave their written informed consent to take part in the study, which was approved by the Research ethics board of the RNQ (Regroupement Neuroimagerie Québec). All procedures were in accordance with the approved guidelines and regulations. Subjects were compensated for their participation.

### Participants

The current report is based on the analyses of 70 healthy young right-handed^44^ adults (mean age = 24.33, SD = 4.65, 50 females). Participation in the study required to be able to perform and learn the motor task. All participants reported no prior history of neurological or psychiatric illness, no brain injury and no addiction to drugs, alcohol or cigarettes (i.e., subjects were non-smokers or occasional smokers). Exclusion criteria included the current or chronic use of medication, any known learning disabilities, and an attention deficit disorder. Only individuals with less than 1 year of formal music training participated in the current study. Professional typists and experienced gamers were excluded as well. All participants had normal quality of sleep, as assessed by the Pittsburgh Sleep Quality Index questionnaire^45^, and reported at least 6 hours of proper nocturnal sleep night before each experimental session.

### Motor sequence learning task

The participants were required to perform a computerized version of the sequential finger tapping task adapted from Karni *et al*. (1995, 1998), as the later has previously been used by different laboratories to characterize different stages of motor sequence learning^17, 24, 27^, including the reconsolidation process of consolidated motor memories in humans^9, 15, 29, 30, 46^. This task was implemented using the Psychophysics Toolbox extensions and implemented in Matlab (The Mathworks, Inc., Natick, MA). Lying supine, participants were instructed to tap a 5-element sequence of finger movements on a 4-key response pad using their left (non-dominant) hand (Fig. 1A). The target sequence was introduced to participants using numbers from 1 to 4 that corresponded to the 4 fingers of their left hand (excluding the thumb) from the little to the index finger respectively. Full explicit introduction of the sequence and instruction to perform the task “as fast and accurate as possible” were given before each experimental session. In case of occasional errors, participants were asked “not to correct errors and to continue the task from the beginning of the sequence”. The session was initiated only after the participant reproduced the target sequence accurately three times in a row, without any error. During each session, periods of rest (25 s) and performance (60 key-presses) were marked by visual stimuli (red and green fixation cross, respectively) presented in the middle of the screen. After completing 60 key presses without feedback (i.e., production of 12 possible 5-element sequences) during each performance block, the color of the stimulus automatically changed from green to red, and subjects were simply required to look at the fixation cross during the rest period. This protocol controlled for the number of movements executed per block to ensure that the same sensory-motor experience with the task was afforded across participants during a particular session.

### Design and Experimental Procedure

The study consisted of three experimental phases, (1) training, (2) memory reactivation with/without an interfering training condition and (3) retest; all of these testing phases being carried out on three consecutive days (Fig. 1B). For each participant, the experimental phases were administered at approximately the same time of day to minimize putative impact of circadian and homeostatic factors on individual performance levels throughout the experiment^47^. During training on the first day (Day1), participants were instructed to perform the sequence 1-4-2-3-1 (T-Seq) for 14 successive performance blocks. Memory reactivation and/or interfering training took place on the second day (Day2). The memory trace for the T-Seq was reactivated using a single performance block (React). This block was also used to test changes in performance during post-training intervals. Behavioral interference was accomplished through an interfering training session that was similar to the initial training on Day1, except that subjects were asked to practice a novel sequence (1-3-2-4-1, Int-Seq). The two sequences, T-Seq and Int-Seq, were composed of identical component movements and were mirror reversed in relation to each other (in terms of order). Thus, the two sequences were matched for the number of movements per digit and differed only in their order. Finally on the last day (Day3), all participants were retested on their performance of the T-Seq using seven blocks of performance.

### Data Analyses

Performance measures were calculated for each participant and, unless otherwise stated, the analyses were designed as mixed repeated measures Analysis of Variance (ANOVA) using individual values as a within-subjects factor and group as a between-subject factor. The results were corrected for non-sphericity violation using the Greenhouse-Geisser adjustment, when appropriate.

Since a single block was available to evaluate performance for the trained sequence on Day2 (i.e., a single block to reactivate the memory trace), for the sake of consistency, performance levels at each time-point of interest were evaluated using data from one performance block only. This approach also minimizes the possibility that performance gains would be biased by additional practice during reactivation and retest sessions. However, there is evidence that performance of the trained motor sequence at the beginning of post-training sessions may be affected by warm-up effects that tend to drastically inflate initial inter-key-press time^25^. Therefore, to account for possible inconsistency in performance and misestimation of actual skill levels, performance measures for each block were assessed using data based on last 30 key-presses only (i.e., equivalent to 6 sequences).

Post-training gains in performance rate were determined using a percentage score based upon performance levels achieved during the last block of the initial training on Day1 (End-T) as a baseline. Accordingly, performance durations during the reactivation block on Day2 and the first retest block on Day3 (React and Retest, respectively) were normalized to baseline, so that better levels of post-training performance, i.e., shorter performance durations, were associated with positive values (Fig. 3, lower right plot). The normalized performance levels calculated for the reactivation block on Day2 constituted the delayed performance gains developed during the first post-training interval. These gains presumably reflect motor memory consolidation processes^11, 16^ and, therefore, are referred as consolidation gains. The normalized performance levels during the first retest block on Day3 constitute overall (total) gains developed by this time since the end of training on Day1. Finally, changes in performance during the post-reactivation interval were determined as a difference between normalized values calculated for the retest on Day3 (i.e., the first retest block) and for the reactivation block on Day2.

Next, we evaluated the rate of performance improvement during continuous experience with the task on Day3. For this purpose, gradient of linear regression (slope) through 4 data-points, corresponding to performance levels achieved by each individual during the first 4 retest blocks were calculated^33^.

Finally, inter-key press intervals (IPIs), i.e., durations between successive key presses, of all correctly completed sequences were extracted for each performance block of interest, being classified according to one of 4 possible transitions between successive elements within a sequence plus an additional transition between the sequences. Average IPIs were then calculated separately for each of five possible transitions excluding any value that was above or beyond two standard deviations from the mean. This procedure resulted in 5-element vectors of IPIs that represent individual IPIs patterns implemented during performance at different time-points of interest (Supplementary Fig. S1). Experience-driven changes in IPIs patterns were assessed using normalized Pearson correlation coefficients calculated between two IPIs patterns at different time-points of interest. Note that the correlation coefficient indicates the degree of similarity between IPIs patterns but do not reflect changes in overall performance rate.

### Data availability

The datasets generated during and/or analyzed during the current study are available from the corresponding author on reasonable request.

## Electronic supplementary material

Supplementary Information
