# Supplementary material for: Re-stepping into the same river: competition problem rather than a reconsolidation failure in an established motor skill
Source: Sci Rep. 2017 Aug 24;7:9406. doi: 10.1038/s41598-017-09677-1 (PMC5570932; doi:10.1038/s41598-017-09677-1)
Supplement: Supplementary file 1 — Supplementary Information [file 41598_2017_9677_MOESM1_ESM.pdf]

Supplementary Information

Re-stepping into the same river: competition problem rather than a reconsolidation failure in an established motor skill

Ella Gabitov, Arnaud Boutin, Basile Pinsard, Nitzan Censor, Stuart M. Fogel, Geneviève Albouy, Bradley R. King, Habib Benali, Julie Carrier, Leonardo G. Cohen, Avi Karni, Julien Doyon

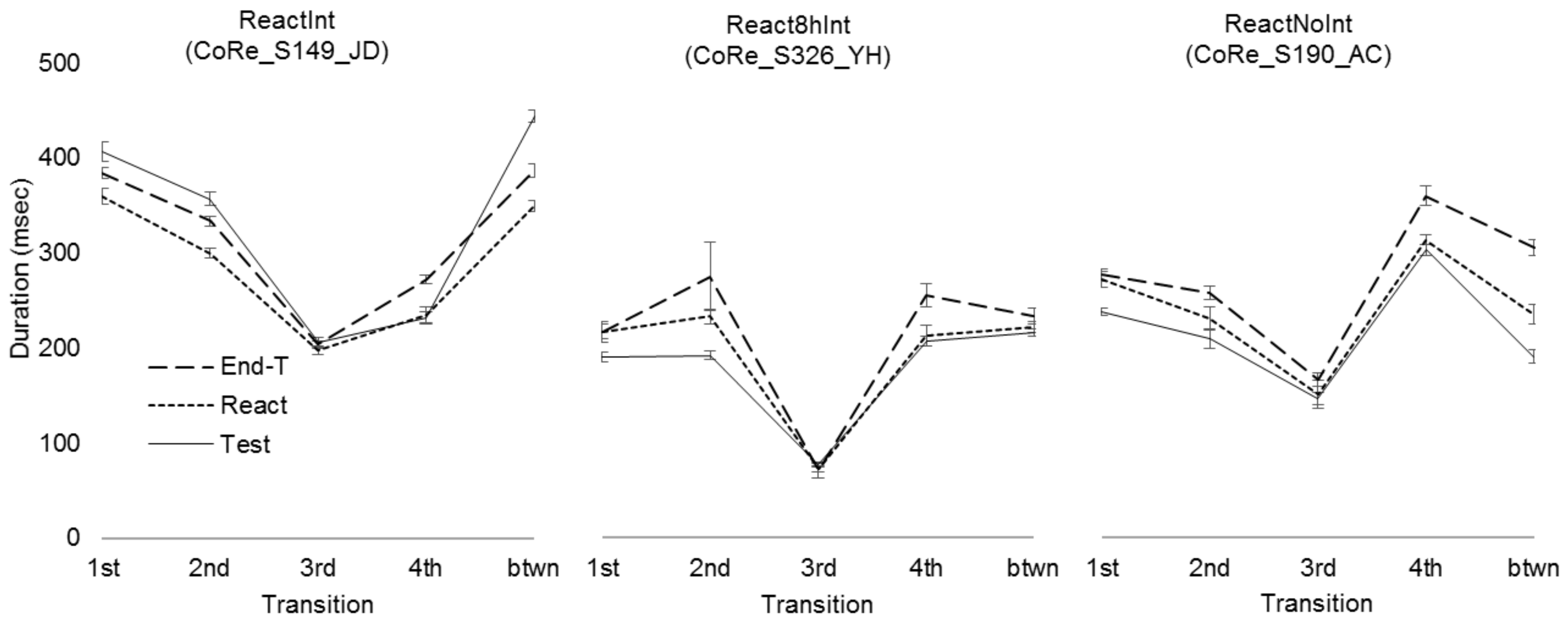

Supplementary Figure S1. Dynamics of individual patterns of inter-key press intervals. Patterns of inter-key press intervals (IPIs) during the last block of training, reactivation block and the first retest-block (End-T, React and Retest respectively) are shown for one representative subject from each group that underwent reactivation of memory trace for the trained sequence on the second experimental day, i.e., ReactInt, React8hInt and ReactNoInt group (left, middle and right panel respectively). Mean time-intervals (i.e., durations) between key presses are plotted for each transition, i.e., a pair of successive elements, of correctly completed sequences (from the 1st to the 4th) as well as between sequences (btwn). Bars – standard error of the mean (s.e.m.).

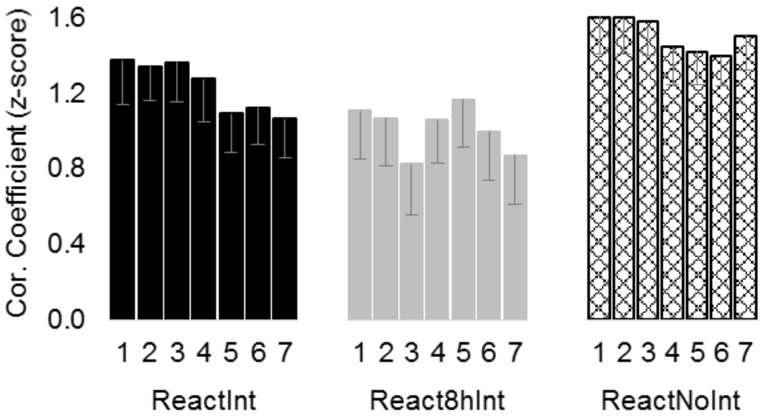

Supplementary Figure S2. Degree of similarity between pattern of inter-key press intervals during the reactivation and retest. Degree of similarity between patterns of inter-key press intervals (IPIs) was assessed based on normalized Pearson correlation coefficients calculated for each individual using the Fisher's z-transformation. Mean normalized Pearson correlation coefficients are shown for each retest-block on Day3 (1 – 7) in ReactInt, React8hInt and ReactInt group. Bars – standard error of the mean (s.e.m.).
